# Food Access and Cardiovascular Outcomes in Metropolitan Atlanta Census Tracts With Residents at Low Risk and High Risk of Cardiovascular Disease: The Morehouse–Emory Cardiovascular Center for Health Equity Study

**DOI:** 10.5888/pcd18.200316

**Published:** 2021-05-06

**Authors:** Shakeria Cohen, Fengxia Yan, Herman Taylor, Mario Sims, Chaohua Li, Arshed A. Quyyumi, Mohamed Mubasher, Tené T. Lewis, Peter Baltrus

**Affiliations:** 1 Cardiovascular Research Institute, Morehouse School of Medicine, Atlanta, Georgia; 2 Community Health and Preventive Medicine, Morehouse School of Medicine, Atlanta, Georgia; 3 Department of Medicine, University of Mississippi Medical Center, Jackson, Mississippi; 4 National Center for Primary Care, Morehouse School of Medicine, Atlanta, Georgia; 5 Emory Clinical Cardiovascular Research Institute, Division of Cardiology, Emory University School of Medicine, Atlanta, Georgia; 6 Department of Epidemiology, Emory University Rollins School of Public Health, Atlanta, Georgia

## Abstract

**Introduction:**

Perceived and actual access to healthy foods may differ in urban areas, particularly among Black people. We assessed the effect of objective and perceived neighborhood food access on self-reported cardiovascular disease (CVD) among Black people living in areas of high risk and low risk for the disease in Atlanta, Georgia. We hypothesized that perceived and objective food access would independently predict self-reported CVD.

**Methods:**

We used survey data from the Morehouse–Emory Cardiovascular (MECA) Center for Health Equity Study. Study participants consisted of 1,402 Black adults, aged 35 to 64, residing in urban Atlanta census tracts with high rates or low rates of CVD. We assessed perceived neighborhood healthy food access by self-reported selection and quality of produce and low-fat food options. We assessed objective food access by the 2015 US Department of Agriculture Food Access Research Atlas. Low access was defined as census tracts with at least 500 people living more than 1 mile from a large food retailer. Self-reported CVD included related conditions and/or procedures. We used multilevel logistic models adjusted for demographic characteristics to examine the association between objective and perceived food access and self-reported CVD.

**Results:**

Overall, self-reported CVD was not significant for perceived (odds ratio = 0.87; 95% CI, 0.59–1.29) or objective (odds ratio = 0.74; 95% CI, 0.48–1.12) healthy food access. Similar results were obtained among adults living in areas with higher-than-expected rates of CVD.

**Conclusion:**

Results of this study suggest the odds for self-reported CVD events were not significantly affected by perceived or objective access to healthy foods.

**Summary**

**What is already known on this topic?**

Black people living in southern states have disproportionate rates of cardiovascular disease (CVD) compared with their White counterparts. Furthermore, living in census tracts with limited access to healthy foods has been associated with higher rates of CVD among this population.

**What is added by this report?**

We sought to better understand the effect of food access on CVD outcomes among a sample of Black adults residing in urban census tracts of Atlanta, Georgia, with low rates and high rates of CVD.

**What are the implications for public health practice?**

Findings from this study could assist in community-level interventions to improve CVD outcomes among Black adults living in areas with high rates of CVD.

## Introduction

Cardiovascular disease (CVD) is the number-one cause of illness and death in the United States (1–3). By the year 2030, an estimated 44% of Americans will be diagnosed with at least 1 form of CVD, comprising $918 billion in annual medical costs (4). Black people have higher rates of CVD mortality and more complications after CVD-related hospitalization compared with other racial/ethnic groups (5–9). Higher prevalence and incidence of CVD among Black people are associated with classic CVD risk factors such as elevated body mass index (BMI), type 2 diabetes, essential hypertension, and smoking (7,9,10). Many of these CVD risk factors are associated with diet; thus, it is important to examine food access in the neighborhoods in which people live (11–13).

A relationship exists between food insecurity, food access, and cardiovascular health (14–17). A neighborhood resident’s perceived access to healthy food may be more important than an objective assessment of access to healthy food. In a study that examined the perception of food access and fast-food consumption among residents of Philadelphia and surrounding counties, negative perceptions of neighborhood fruits and vegetables, travel time to food retailers, and food quality were significantly associated with increased fast-food intake and decreased produce consumption among Black residents, compared with non-Hispanic White residents, who perceived better access to healthy foods (18).

The role of food access in CVD and health inequality is unclear. The overall risk of CVD among the Black population is 3 times higher than among the White population (19). Because residential location is associated with social position, racial composition, and health outcomes, examining attributes of food access in neighborhoods could help to advance health equity (15,17,19,20). To our knowledge, objectively measured food access and perceived food access as predictors of CVD among Black people have not been studied in a single study population. Our study sought to describe the relationship between both objectively measured and perceived food access and self-reported CVD among Black adults residing in urban areas in Atlanta, Georgia. We hypothesized that objectively measured food access and perceived food access would independently predict self-reported CVD in our study population.

## Methods

We used data from surveys administered from August through October 2016 by the Morehouse–Emory Cardiovascular (MECA) Center for Health Equity Study. The MECA study was designed as a retrospective, cross-sectional study to examine the cardiovascular health of Black adults aged 35 to 64; participants were recruited by using a random-digit–dialing system from census tracts of 36 counties in metropolitan Atlanta, Georgia (7,15). We used census tracts as a proxy for neighborhoods. We assessed census tract data from 2010 through 2014 for CVD–at-risk tracts (n = 121) (those with higher-than-expected rates of adverse CVD outcomes) and CVD-resilient tracts (n = 106) (tracts with lower-than-expected rates of adverse CVD outcomes) by using distributions of residuals from census-tract–level regression models for each outcome. Models were adjusted for household income, age, and sex. Adverse CVD outcomes were cardiovascular mortality, emergency department visits, and CVD-related hospitalizations. Details on the selection of census tracts and study participants are available elsewhere (7,15). Data collection for the MECA study has been completed, but analysis is ongoing. We included in these analyses only study participants living in urban tracts, identified according to 2010 US Census classifications; we excluded 31 participants residing in rural areas. Of 1,402 participants, 683 resided in CVD-resilient census tracts and 719 resided in CVD–at-risk census tracts.

### Statistical hypothesis

Analyses were conducted to test the association between self-reported CVD (dependent variable/ outcome measure) and perceived and objective food access (analyses main independent variable).

### Independent variables

**Perceived neighborhood healthy food access.** Self-reported perception of neighborhood healthy food access was assessed via a neighborhood health questionnaire, validated for studying cardiovascular health (21). The 3 food-access–related items were 1) “A large selection of fresh fruits and vegetables is available in my neighborhood,” 2) “The fresh fruits and vegetables in my neighborhood are of high quality,” and 3) “A large selection of low-fat foods are available in my neighborhood.” Answers were given a 5-point Likert scale: 1, strongly agree; 2, agree; 3, neither agree nor disagree; 4, disagree; 5, strongly disagree. A composite score (range, 3–15) was created by summing the responses to each item. The higher the score, the lower one’s perception of the neighborhood’s healthy food access. A score of ≤12 was defined as having a perception of a high level of healthy food access; a score of 13 to 15 was defined as having a perception of low level of healthy food access.

**Objectively measured food access.** We cross-referenced data from the 2015 US Department of Agriculture’s Food Access Research Atlas with census tract data from the MECA study (22). The Food Access Research Atlas classifies urban census tracts as having low levels of access to healthy foods when 500 or more people or 33% of the census tract population resides 1 mile or more from a large grocery store, supercenter, or supermarket (22).

### Dependent/outcome variable

We classified respondents as having CVD (yes/no) if they answered yes to being diagnosed with or having any of the following: myocardial infarction, angina, atrial fibrillation, congestive heart failure, coronary artery bypass, stroke, defibrillation, balloon angioplasty, heart valve replacement, pacemaker implant, or heart surgery.

### Cofounders and covariates

We used dichotomized variables (yes/no) for the following: BMI more than 25 (based on self‐reported height and weight, kg/m^2^), diabetes, high cholesterol, and hypertension. Smoking status was grouped into 4 categories: current smoker, quit in the last year, quit more than 1 year, and never smoked. We created a dichotomized variable for individual-level socioeconomic status (SES) from self-reported income and educational attainment. We defined low individual-level SES as having an annual household income of $50,000 or less or, when data were missing, having a high school diploma or less. Employment was grouped into 4 categories: employed full-time or part-time, not working/unemployed, a homemaker; and retired. Marital status was grouped into 3 categories: married, divorced/separated/widowed, and never married/unmarried. We also used data from the 2010 US Census to assess median annual household income.

### Statistical models

Univariate analyses were used to determine significant differences for continuous variables, 2-sample *t* tests for normally distributed variables, and a Wilcoxon rank-sum nonparametric test for nonnormally distributed variables. We used a χ^2^ test to compare proportions for categorical variables. Multivariate analyses were used to determine the association between self-reported CVD and perceived and objectively measured access to healthy foods while adjusting for confounding variables. The generalized linear mixed models also accounted for clustering by census tract. Model 1 was unadjusted for perceived and objectively measured food access on self-reported CVD. Model 2 was adjusted for CVD–at-risk and CVD-resilient neighborhoods. Model 3 was mutually adjusted for both independent variables and CVD–at-risk and CVD-resilient neighborhoods. An interaction term for objectively measured food access and perceived food access was not significant. Models 4 and 5 included Model 2 plus age, BMI, sex, marital status, individual-level SES, employment, and census-tract median household income. Model 6 was adjusted for CVD risk and behaviors: diabetes, high cholesterol, hypertension, and smoking. The outcome variable was self-reported CVD-related conditions and procedures. A final analysis included Model 6 with mutual adjustments for objectively measured food access and perceived food access. We first assessed models for an interaction between the main predictor variables and CVD–at-risk and CVD-resilient census tracts and found no significant interactions (ie, the effect of objectively measured and perceived food access did not differ by whether a census tract was at risk or resilient). Therefore, we adjusted Models 2 through 6 for census-tract CVD risk status (at risk or resilient). We used SAS version 9.4 (SAS Institute, Inc) for all analyses; significance was defined as *P* < .05. In addition, we created a map that shows objectively measured levels of food access, by 2010 census tract boundaries, in Atlanta and the number of survey respondents in each census tract; we created the map by using ArcGIS Pro (Esri).

## Results

Census tracts with high levels of objectively measured food access tended to be close to the center of Atlanta, whereas census tracts with low levels tended to be in the surrounding areas (Figure).

**Figure:**
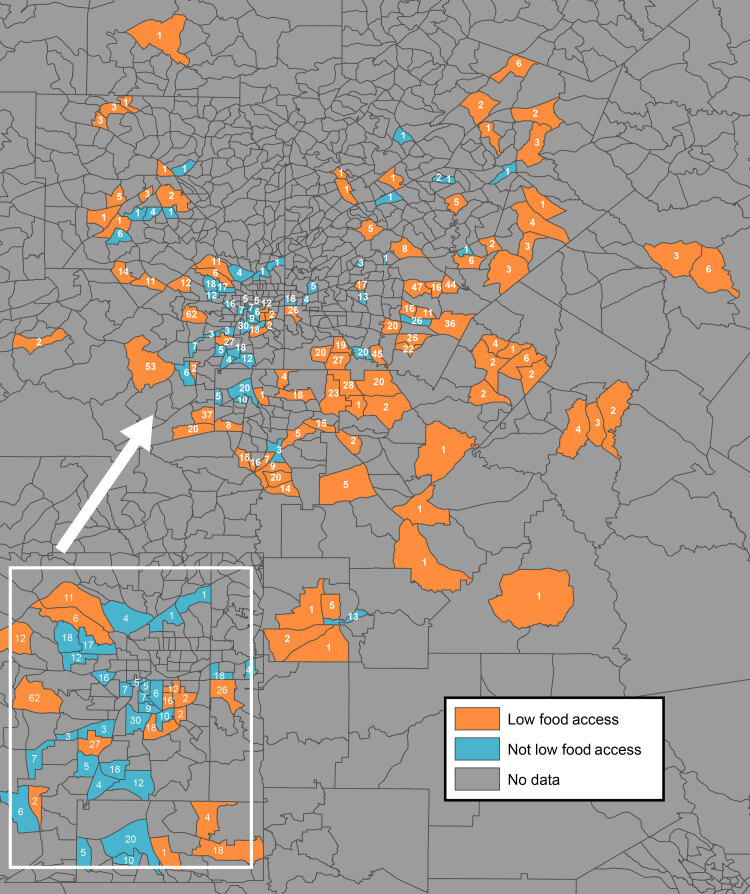
Objectively measured levels of food access, by 2010 census tract boundaries, in metropolitan Atlanta, Georgia. Only census tracts in which participants in the Morehouse–Emory Cardiovascular Center for Health Equity Study (indicated by the numbers inside census tracts) resided were examined for food access. “Low food access” refers to census tract areas that had objectively measured low levels of access to healthy foods, and “not low food access” refers to census tracts areas that had objectively measured high levels of access to healthy foods. The US Department of Agriculture Food Access Research Atlas classifies urban census tracts as having low levels of access to healthy foods when ≤500 people or 33% of the census tract population resides 1 mile or more from a large grocery store, supercenter, or supermarket (22). Inset shows the city of Atlanta.

The mean (SD) age of the study population was 51.6 (10.2) years; 61.3% were women (Table 1). We found no difference by age or BMI between perceived and objectively measured food access. Participants who perceived low levels of neighborhood healthy food access were significantly more likely than participants who perceived high levels to be married (46.4% vs 39.4%; *P* = .03), live in CVD-resilient census tracts (54.2% vs 42.0%; *P* < .001), be employed full or part time (63.9% vs 60.6%; *P* = .02), and have high individual-level SES (62.6% vs 53.3%; *P* < .001) and higher census-tract median household income (*P* < .001) (Table 1). Moreover, participants residing in census tracts with low levels of objectively measured healthy food access were more likely than participants in census tracts with high levels to be married (47.8% vs 30.7%; *P* < .001), reside in CVD-resilient census tracts (57.8% vs 23.2%; *P* < .001), be employed full time or part time (64.6% vs 56.5%; *P* = .001), and to have high individual-level SES (62.6% vs 46.8%; *P* < .001) and higher census-tract median household income (*P* < .001) (Table 1).

**Table 1:** Characteristics of the Study Population, by Neighborhood Food Access, Morehouse–Emory Cardiovascular (MECA) Center for Health Equity Study, 2016^a^

Characteristic	All (N = 1,402)	Perceived Neighborhood Healthy Food Access^b^ (n = 1,362)		*P* Value^c^	Objectively Measured Healthy Food Access^d^ (n = 1,402)		*P* Value^c^
		Low	High		Low	High	
**Age**	51.6 (10.2)	52.0 (10.0)	51.1 (10.5)	.11	51.7 (10.1)	51.3 (10.5)	.56
**Sex**							
Male	542 (38.7)	301 (38.7)	230 (39.4)	.79	408 (39.4)	134 (36.5)	.33
Female	860 (61.3)	477 (61.3)	354 (60.6)		627 (60.6)	233 (63.5)	
**Body mass index, mean (SD)**	30.3 (6.9)	30.0 (6.4)	30.7 (7.4)	.07	30.2 (7.0)	30.5 (7.8)	.47
**Marital status**							
Married	604 (43.3)	360 (46.4)	228 (39.4)	.03	492 (47.8)	112 (30.7)	<.001
Divorced/separated/widowed	404 (29.0)	210 (27.1)	182 (31.4)		277 (26.9)	127 (34.8)	
Never married/unmarried	386 (27.7)	205 (26.5)	169 (29.2)		260 (25.3)	126 (34.5)	
**Neighborhood CVD risk^e^**							
Resilient	683 (48.7)	422 (54.2)	245 (42.0)	<.001	598 (57.8)	85 (23.2)	<.001
At risk	719 (51.3)	356 (45.8)	339 (58.0)		437 (42.2)	282 (76.8)	
**Employment status**							
Employed full time or part time	869 (62.5)	494 (63.9)	350 (60.6)	.02	664 (64.6)	205 (56.5)	.001
Not working or unemployed	134 (9.6)	59 (7.6)	73 (12.6)		82 (8.0)	52 (14.3)	
Homemaker	74 (5.3)	42 (5.4)	28 (4.8)		50 (4.9)	24 (6.6)	
Retired	314 (22.6)	178 (23.0)	127 (22.0)		232 (22.5)	82 (22.6)	
**Individual-level SES^f^**							
Low (annual household income ≤$50,000)	578 (41.5)	289 (37.4)	271 (46.7)	<.001	385 (37.4)	193 (53.2)	<.001
High (annual household income >$50,000)	815 (58.5)	484 (62.6)	309 (53.3)		645 (62.6)	170 (46.8)	
**Census-tract median household income, $^g^**	54,443	57,980	49,820	<.001	60,070	38,580	<.001
**Cardiovascular disease^h^**							
Yes	116 (8.3)	61 (7.8)	52 (8.9)	.48	79 (7.6)	37 (10.1)	.14
No	1,286 (91.7)	717 (92.2)	532 (91.1)		956 (92.4)	330 (89.9)	
**Diabetes**							
Yes	243 (17.3)	123 (15.8)	113 (19.4)	.09	174 (16.8)	69 (18.8)	.39
No	1159 (82.7)	655 (84.2)	471 (80.6)		861 (83.2)	298 (81.2)	
**High cholesterol**							
Yes	324 (23.1)	178 (22.9)	140 (24.0)	.64	242 (23.4)	82 (22.3)	.69
No	1,078 (76.9)	600 (77.1)	444 (76.0)		793 (76.6)	285 (77.7)	
**Hypertension**							
Yes	635 (45.3)	343 (44.1)	272 (46.6)	.36	460 (44.4)	175 (47.7)	.28
No	767 (54.7)	435 (55.9)	312 (53.4)		575 (55.6)	192 (52.3)	
**Smoking**							
Current smoker	193 (13.8)	95 (12.2)	91 (15.6)	.24	126 (12.2)	67 (18.3)	.008
Quit within past year	36 (2.6)	20 (2.6)	16 (2.7)		26 (2.5)	10 (2.7)	
Quit more than a year	236 (16.8)	127 (16.3)	102 (17.5)		167 (16.1)	69 (18.8)	
Never smoked	937 (66.8)	536 (68.9)	375 (64.2)		716 (69.2)	221 (60.2)	

Abbreviations: CVD, cardiovascular disease; SES, socioeconomic status.

a Data collected from a survey of Black adults aged 35–64 recruited by using a random-digital–dialing system from census tracts in metropolitan Atlanta, Georgia (7,15). Numerical values are expressed as mean (SD) and categorical variables as frequency (percentage). All values were self-reported unless indicated otherwise.

b Participants responded to 3 food access–related items: 1) “A large selection of fresh fruits and vegetables is available in my neighborhood,” 2) “The fresh fruits and vegetables in my neighborhood are of high quality,” and 3) “A large selection of low-fat foods are available in my neighborhood.” Answers were given a 5-point Likert scale: 1, strongly agree; 2, agree; 3, neither agree nor disagree; 4, disagree; 5, strongly disagree. A composite score (range, 3–15) was created by summing the responses to each item. The higher the score, the lower one’s perception of the neighborhood’s healthy food access. A score of ≤12 was defined as having a perception of a high level of healthy food access.

c *P* values determined by χ^2^ for categorical variables and *t* test for continuous variables.

d Cross-referenced data from the 2015 US Department of Agriculture’s Food Access Research Atlas with census tract data from the MECA study. The Food Access Research Atlas classifies urban census tracts as having low levels of access to healthy foods when ≥500 people or 33% of the census tract population resides ≥1 mile from a large grocery store, supercenter, or supermarket (22).

e Census tract data for 2010–2014 assessed for higher-than-expected (at risk [n = 121 census tracts] and lower-than-expected (resilient [n = 106 census tracts]) rates of adverse CVD outcomes (cardiovascular mortality, emergency department visits, and CVD-related hospitalizations).

f When data on income were missing, low SES was defined as having ≤high school diploma.

g Data source: 2010 US Census.

h Myocardial infarction, angina, atrial fibrillation, congestive heart failure, coronary artery bypass, stroke, defibrillation, balloon angioplasty, heart valve replacement, pacemaker implant, or heart surgery.

Differences in self-reported CVD risk factors (diabetes, high cholesterol, and hypertension) and smoking status were not significant between groups with high and low levels of perceived neighborhood healthy food access. We observed similar results for objectively measured healthy food access for these factors, except that among smokers, 18.3% resided in objectively measured high-access areas and 12.2% resided in low-access areas (*P* = .008) (Table 1). The association between objectively measured food access and perceived food access was not significant.

In the unadjusted multilevel logistic Model 1, we found no differences between high and low for perceived (odds ratio [OR] = 0.87; 95% CI, 0.59–1.29) or objectively measured healthy food access (OR = 0.74; 95% CI, 0.48–1.12) and self-reported CVD (Table 2). After multilevel adjustments for CVD–at-risk neighborhoods and CVD-resilient neighborhoods (Table 2, Model 2), perceived (OR = 0.87; 95% CI, 0.59–1.30) and objectively measured (OR = 0.72; 95% CI, 0.46–1.12) food access did not change significantly. Likewise, after adjustments for both food access variables (Table 2, Model 3), neither perceived access (OR = 0.89; 95% CI, 0.60–1.31) nor objectively measured access (OR = 0.73; 95% CI, 0.47–1.15) altered the relationship for self-reported CVD. After further adjustments for demographic characteristics and BMI (Table 2, Models 4 and 5), the results shifted even closer to the null. In the fully adjusted model (Table 2, Model 6) for CVD risk factors and smoking status, we found no significant association between perceived or objectively measured healthy food access on self-reported CVD. 

**Table 2:** Odds Ratios of Self-Reported Cardiovascular Disease, by Perceived and Objectively Measured Levels of Access to Neighborhood Healthy Food, Morehouse–Emory Cardiovascular (MECA) Center for Health Equity Study, 2016^a^

Variable	Cardiovascular Disease^b^		Odds Ratio (95% CI)					
	Yes	No	Model 1^c^	Model 2^d^	Model 3^e^	Model 4^f^	Model 5^g^	Model 6^h^
**Perceived neighborhood healthy food access^i^**								
Intraclass correlation coefficient	—		0	0	0	0	0	0.0316
Low	61 (7.8)	717 (92.2)	0.87 (0.59–1.29)	0.87 (0.59–1.30)	0.89 (0.60–1.31)	0.89 (0.58–1.37)	0.93 (0.60–1.43)	0.97 (0.62–1.52)
High	52 (8.9)	532 (91.1)						
**Objectively measured healthy food access^j^**								
Intraclass correlation coefficient	—		0.0027	0.0033	0	0.0049	0.044	0.0420
Low	79 (7.6)	956 (92.4)	0.74 (0.48–1.12)	0.72 (0.46–1.12)	0.73 (0.47–1.15)	0.92 (0.57–1.49)	1.06 (0.63–1.79)	1.04 (0.60–1.78)
High	37 (10.1)	330 (89.9)						

a Data collected from a survey of Black adults aged 35 to 64 recruited by using a random-digital–dialing system from census tracts in metropolitan Atlanta, Georgia (7,15).

b Self-reported myocardial infarction, angina, atrial fibrillation, congestive heart failure, coronary artery bypass, stroke, defibrillation, balloon angioplasty, heart valve replacement, pacemaker implant, or heart surgery.

c Model 1 = unadjusted.

d Model 2 = Model 1 + adjustment for CVD–at-risk (higher-than-expected) and CVD-resilient (lower-than-expected) neighborhoods. Census tract data for 2010–2014 assessed for rates of at risk (n = 121 census tracts) and resilient (n = 106 census tracts) adverse CVD outcomes.

e Model 3 = Model 2 + mutually adjusted for perceived food access and objectively measured food access.

f Model 4 = Model 2 +adjustment for age, sex, marital status, body mass index, individual-level socioeconomic status, and employment status.

g Model 5 = Model 4 + adjustment for community income.

h Model 6 = Model 5 + adjustment for diabetes, high cholesterol, hypertension, smoking, and employment status.

i Participants responded to 3 food access–related items: 1) “A large selection of fresh fruits and vegetables is available in my neighborhood,” 2) “The fresh fruits and vegetables in my neighborhood are of high quality,” and 3) “A large selection of low-fat foods are available in my neighborhood.”

j Cross-referenced data from the 2015 US Department of Agriculture’s Food Access Research Atlas with census tract data from the MECA study. The Food Access Research Atlas classifies urban census tracts as having low levels of access to healthy foods when ≥500 people or 33% of the census tract population resides ≥1 mile from a large grocery store, supercenter, or supermarket (22).

Many unadjusted associations for the variables examined were significant (Table 3). In the fully adjusted model, being employed full time or part time (OR = 0.35; 95% CI, 0.19–0.63) protected against self-reported CVD. In addition, high cholesterol (OR = 2.86; 95% CI, 1.81–4.54) and hypertension (OR = 2.17; 95% CI, 1.20–3.91) were significantly associated with self-reported CVD (Table 3).

**Table 3:** Final Model Indicating the Association Between Neighborhood Healthy Food Access and Other Risk Factors of Self-Reported Cardiovascular Disease^a^, Morehouse–Emory Cardiovascular (MECA) Center for Health Equity Study, 2016^b^

Characteristic	Unadjusted	Full Model^c^
	Odds Ratio (95% CI)	Odds Ratio (95% CI)
**Perceived food access^d^**	0.87 (0.59–1.29)	0.97 (0.62–1.53)
**Objectively measured food access^e^**	0.74 (0.48–1.12)	0.98 (0.57–1.70)
**Age**	1.08 (1.06–1.11)	1.03 (0.99–1.06)
**Sex**	1.01 (0.67–1.50)	0.64 (0.40–1.05)
**Body mass index**	1.04 (1.02–1.07)	1.01 (0.98–1.04)
**Marital status**		
Married	0.76 (0.45–1.28)	0.54 (0.29–1.02)
Divorced/separated/widowed	1.81 (1.11–2.95)	0.87 (0.48–1.58)
Never married/unmarried	1 [Reference]	1 [Reference]
**Neighborhood CVD risk^f^**		
Resilient	0.98 (0.66–1.44)	1.30 (0.80–2.10)
At risk	1 [Reference]	1 [Reference]
**Employment**		
Employed full time or part time	0.16 (0.10–0.25)	0.35 (0.19–0.63)
Not working or unemployed	0.68 (0.38–1.22)	0.55 (0.27–1.12)
Homemaker	0.92 (0.46–1.82)	1.26 (0.54–2.95)
Retired	1 [Reference]	1 [Reference]
**Individual-level SES**		
Low (annual household income ≤$50,000)	1 [Reference]	1 [Reference]
High (annual household income >$50,000)	3.02 (2.00–4.56)	1.44 (0.87–2.50)
**Census-tract median household income^g^**	0.54 (0.9–0.64)	0.54 (0.15–1.97)
**Diabetes**	3.50 (2.31–5.31)	1.60 (0.97–2.65)
**High cholesterol**	5.02 (3.32-7.58)	2.86 (1.81–4.54)
**Hypertension**	5.35 (3.32–8.63)	2.17 (1.20–3.91)
**Smoking**		
Current smoker	2.47 (1.50–4.07)	1.89 (1.06–3.38)
Quit within past year	2.01 (0.68–5.94)	1.46 (0.45–4.79)
Quit more than 1 year	2.01 (1.23–3.29)	1.16 (0.66–2.03)
Never smoked	1 [Reference]	1 [Reference]

Abbreviations: CVD, cardiovascular disease; SES, socioeconomic status.

a Self-reported myocardial infarction, angina, atrial fibrillation, congestive heart failure, coronary artery bypass, stroke, defibrillation, balloon angioplasty, heart valve replacement, pacemaker implant, or heart surgery.

b Data collected from a survey of Black adults aged 35 to 64 recruited by using a random-digital–dialing system from census tracts in metropolitan Atlanta, Georgia (7,15).

c Adjusted for all variables simultaneously.

d Participants responded to 3 food access–related items: 1) “A large selection of fresh fruits and vegetables is available in my neighborhood,” 2) “The fresh fruits and vegetables in my neighborhood are of high quality,” and 3) “A large selection of low-fat foods are available in my neighborhood.”

e Cross-referenced data from the 2015 US Department of Agriculture’s Food Access Research Atlas with census tract data from the MECA study. The Food Access Research Atlas classifies urban census tracts as having low levels of access to healthy foods when ≥500 people or 33% of the census tract population resides ≥1 mile from a large grocery store, supercenter, or supermarket (22).

f Census tract data from 2010 through 2014 assessed for CVD–at-risk tracts (n = 121) (those with higher-than-expected rates of adverse CVD outcomes) and CVD-resilient tracts (n = 106) (tracts with lower-than-expected rates of adverse CVD outcomes).

g Data source: 2010 US Census.

## Discussion

We examined whether objectively measured and perceived neighborhood access to healthy food were associated with self-reported CVD prevalence among Black adults living in urban areas of Atlanta, Georgia. We hypothesized that both measures of healthy food access would be independently associated with higher rates of self-reported CVD. Our overall results suggested that the odds of self-reported CVD were not significantly affected by either measure. Even after adjusting for differences in age, sex, BMI, marital status, employment, individual-level SES, and census-tract median household income, neither measure was associated with self-reported CVD. In the fully adjusted models that included CVD risk factors and smoking status, we found no significant effect of either measure on self-reported CVD. However, we did find that employment status protected against self-reported CVD and that high cholesterol and hypertension were significantly associated with increased odds of self-reported CVD.

To our knowledge, this is the first study to examine an association between objectively measured and perceived access to healthy food and the prevalence of CVD in a sample of Black adults. Our approach was novel in that we examined both perception of healthy food access and objectively measured healthy food access. Interestingly, we did not observe a significant association between these 2 measures among our study participants. Furthermore, our findings suggest that lack of healthy food access close to home is not a significant determinant of cardiovascular health. This unpredicted finding raises questions about the commonly used definition of food access and the possible factors that influence CVD outcomes among Black adults, particularly in metropolitan Atlanta and perhaps in other regions of the United States.

Several studies reported on the positive association between neighborhood attributes and poor cardiovascular health (7,15,21,23). It has become axiomatic that residential proximity to healthy foods improves the likelihood of good cardiovascular health. In the Multi-Ethnic Study of Atherosclerosis (MESA) neighborhood study, one of the largest multiracial prospective studies to date (24), researchers investigated the pervasiveness and advancement of subclinical CVD among 6,500 men and women from diverse racial/ethnic backgrounds (24). The study participants were followed for 7 years for incidence of CVD-related conditions, including myocardial infarction, stroke, and coronary artery disease. A review by Diez-Roux et al summarized findings from MESA and similar studies, outlining best approaches for assessing the influence of neighborhood environments on CVD risk (25). That study reported a positive association between living in census tracts with better access to healthy foods and lower BMI; it also reported a lower prevalence of hypertension in these census tracts than in census tracts with socioeconomically disadvantaged residents and limited healthy food access (25).

Although our study may seem to contradict previous studies and our results may seem to be counterintuitive, several studies conducted in Atlanta and other southern states corroborate our results. Gaglioti et al tested whether premature CVD mortality was associated with having inadequate access to healthy foods and restricted walkability in census tracts around Atlanta (1). They found that having low levels of access to healthy foods and a nonfriendly walking environment increased the number of untimely CVD-related deaths. However, the associations were not significant when census tracts comprising only Black residents were added to the model. In their fully adjusted model, which included community poverty level and Black residents, having better food access in one’s neighborhood was not significantly related to untimely CVD deaths (1). Kelli et al examined whether living in food deserts influenced CVD risk among Atlanta-area participants of the META-Health and Predictive Health studies and found that living in areas with limited access to nutritious foods increased CVD risk, although the risk of CVD was mainly driven by area income, not limited access to healthy foods (14). A prospective study among patients undergoing cardiac cauterization at Emory Biobank found that after following participants for 3.2 years for occurrence of myocardial infarction or death due to CVD, living in areas with low access to healthy foods was linked to higher risk of myocardial infarction; however, area income, not unhealthy food access, was the cause of worse health outcomes (26).

A study by Freedman and Bell assessed the difference between perception and actual healthy food access among residents living in communities around Nashville, Tennessee. Through self-reported surveys, the authors assessed healthy and nonhealthy foods available for purchase in neighborhood food markets and perception of food accessibility (12). Participants were asked to rate their view of the convenience of purchasing fresh and quality produce. The authors found no significant difference between a person’s perception of healthy foods in one’s neighborhood and healthy foods available for purchase (12). Reports from these studies and others highlight the nuanced understanding of healthy neighborhood food access and its effect on health.

Topel et al used MECA data to examine differences in neighborhood attributes and psychosocial factors among residents of census tracts at high and low risk of CVD (7). They found that residents of high-risk census tracts reported poorer healthy neighborhood food access than residents of low-risk tracts (7). Kaiser and colleagues used data from the MESA project to study the association between community attributes and the public environment on risk of hypertension. The authors reported a lower prevalence of hypertension in areas with better healthy food access (27). Furthermore, limited access to healthy foods and an unfriendly walkable community were highly correlated with incidence of hypertension. Another study that used data from MESA to examine neighborhood characteristics and cardiovascular health reported that advantageous neighborhood attributes — healthy food options, walkability, and high SES — directly correlated with decreased risk of CVD (28). A study of 2 predominantly Black neighborhoods in Pittsburgh, Pennsylvania, reported that a new full-service grocery store improved perception of the availability of healthy foods in the neighborhood and found healthier dietary behaviors among residents. However, the authors concluded that their findings were unrelated to shopping at the full-service grocery store, suggesting that perception plus access may have influenced changes in dietary behaviors, not access alone (29).

Although previous studies add to the overall scientific literature, they mostly compared a racially and socioeconomically diverse group of White and Black people across a wide range of ages. Our study focused solely on a population of Black adults living in areas of low and high risk of CVD and the relationship between CVD and healthy food access. Our unexpected results raise questions about how Black residents of cities like Atlanta procure healthy foods. Surprisingly, we found that Black adults residing in areas of objectively measured low healthy food access were highly educated and earned more household income than Black adults residing in areas of objectively measured high healthy food access. Interestingly, our maps showed that low-access census tracts were mostly located outside the city, whereas high-access census tracts were largely in the city. Although convenience of healthy food access is an obvious social good, our study suggests that higher SES among Black residents living in Atlanta may easily overcome the barrier of physical distance, which may have reduced the importance of this variable in our analysis. Furthermore, lower-SES residents of the inner city may live close to upscale food outlets, but they may not be able to afford to shop in them routinely (or are pushed toward cheaper, less healthy foods). Interventions aimed at improving food access in food deserts may not improve healthy food consumption, but interventions that promote healthy dietary behaviors could help decrease the prevalence of preventable diseases such as CVD among Black people while closing the gap on CVD-related health disparities.

Our study has several limitations. First, our study was cross-sectional; therefore, we were unable to establish temporality between the predictor (food access) and the outcome (self-reported CVD). Although it is not likely that having CVD would result in respondents choosing neighborhoods with less food access, as the results suggest, the association with perceived food access may be more problematic and may lead one to become more conscious of what constitutes healthy food choices. Future prospective studies of this population could better establish the temporality of any observed associations. Second, people who chose to participate in the survey may not have represented the general population of the neighborhoods we sampled; past analysis of the MECA study showed study participants to be healthier and have a higher SES than the general population in the census tracts from which they were selected. Third, we relied on self-reported outcomes; respondents may have had undiagnosed CVD or may not have recalled a diagnosis, although the events we used to define CVD are generally well-remembered by respondents. Fourth, it was not possible to determine whether a respondent’s perception of food access truly reflected the accessibility of food in their neighborhood or reflected only the neighborhood stores they shopped in (15,29). “Perceived” access is how the respondents perceived food accessibility in their neighborhood; it cannot be used as a proxy for objectively measured food access. Fifth, we acknowledge that many variables contribute to neighborhood food access, and we included only a few. Additional studies are warranted to further understand the complexity of the definition of neighborhood healthy food access. Finally, our data were not weighted to represent the underlying population; thus, generalizations cannot be made to the entire metropolitan Atlanta area or to other areas of the United States.

Despite these limitations, our study has strengths. We focused on a group of Black adults from a wide spectrum of SES and residing in a large metropolitan city. Although our findings are limited to one metropolitan city and may not be generalizable to the general population, they help to clarify factors that increase the risk of, or promote resilience to, poor health among Black residents across a range of census tracts. We showed that neither objectively measured healthy food access nor perceived healthy food access was independently associated with self-reported CVD among Black adults residing in urban areas of metropolitan Atlanta. Surprisingly, people living in areas of objectively measured high healthy food access had higher odds of reporting CVD than people living in low-access neighborhoods. Even so, the data were not weighted, and our results are applicable only to our study participants. Studies in other urban areas are needed to determine whether the observations are generalizable to Black people living in other areas.
